# Axillary Microbiota Is Associated with Cognitive Impairment in Parkinson’s Disease Patients

**DOI:** 10.1128/spectrum.02358-21

**Published:** 2022-02-09

**Authors:** Muzaffer Arikan, Zeynep Yildiz, Tugce Kahraman Demir, Nesrin H. Yilmaz, Aysu Sen, Lutfu Hanoglu, Suleyman Yildirim

**Affiliations:** a Regenerative and Restorative Medicine Research Center (REMER), Research Institute for Health Sciences and Technologies (SABITA), Istanbul Medipol University, Istanbul, Turkey; b Graduate Program in Neuroscience, Istanbul Medipol University, Istanbul, Turkey; c Programme of Electroneurophysiology, Vocational School, Biruni University, Istanbul, Turkey; d Department of Neurology, Istanbul Medipol University Training Hospital, Istanbul, Turkey; e Department of Neurology, Bakirkoy Research and Training Hospital for Psychiatric and Neurological Diseases, Istanbul, Turkey; f Department of Medical Microbiology, International School of Medicine, Istanbul Medipol University, Istanbul, Turkey; Huazhong University of Science and Technology

**Keywords:** axillary microbiota, Parkinson’s disease, cognitive impairment, dementia, armpit, 16S sequencing, armpit microbiota, skin microbiota

## Abstract

Cognitive impairment (CI) is among the most common non-motor symptoms of Parkinson’s disease (PD), with a substantially negative impact on patient management and outcome. The development and progression of CI exhibits high interindividual variability, which requires better diagnostic and monitoring strategies. PD patients often display sweating disorders resulting from autonomic dysfunction, which has been associated with CI. Because the axillary microbiota is known to change with humidity level and sweat composition, we hypothesized that the axillary microbiota of PD patients shifts in association with CI progression, and thus can be used as a proxy for classification of CI stages in PD. We compared the axillary microbiota compositions of 103 PD patients (55 PD patients with dementia [PDD] and 48 PD patients with mild cognitive impairment [PD-MCI]) and 26 cognitively normal healthy controls (HC). We found that axillary microbiota profiles differentiate HC, PD-MCI, and PDD groups based on differential ranking analysis, and detected an increasing trend in the log ratio of *Corynebacterium* to *Anaerococcus* in progression from HC to PDD. In addition, phylogenetic factorization revealed that the depletion of the *Anaerococcus, Peptoniphilus*, and *W5053* genera is associated with PD-MCI and PDD. Moreover, functional predictions suggested significant increases in myo-inositol degradation, ergothioneine biosynthesis, propionate biosynthesis, menaquinone biosynthesis, and the proportion of aerobic bacteria and biofilm formation capacity, in parallel to increasing CI. Our results suggest that alterations in axillary microbiota are associated with CI in PD. Thus, axillary microbiota has the potential to be exploited as a noninvasive tool in the development of novel strategies.

**IMPORTANCE** Parkinson's disease (PD) is the second most common neurodegenerative disease. Cognitive impairment (CI) in PD has significant negative impacts on life quality of patients. The emergence and progression of cognitive impairment shows high variability among PD patients, and thus requires better diagnostic and monitoring strategies. Recent findings indicate a close link between autonomic dysfunction and cognitive impairment. Since thermoregulatory dysfunction and skin changes are among the main manifestations of autonomic dysfunction in PD, we hypothesized that alterations in the axillary microbiota may be useful for tracking cognitive impairment stages in PD. To our knowledge, this the first study characterizing the axillary microbiota of PD patients and exploring its association with cognitive impairment stages in PD. Future studies should include larger cohorts and multicenter studies to validate our results and investigate potential biological mechanisms.

## INTRODUCTION

Parkinson’s disease (PD) is the second most common neurodegenerative disorder after Alzheimer’s disease, with a worldwide prevalence of more than 6 million affected individuals in 2016. A considerable increase in the disease incidence is estimated in future decades (1). Treatment options for PD are limited as the underlying pathophysiological molecular mechanisms are still poorly understood. As a complex and heterogenous disease, PD is clinically characterized by the progressive reduction of both motor and non-motor functions. Cognitive impairment (CI) and autonomic dysfunction are among the most common non-motor symptoms of PD (2).

CI progressively develops on a spectrum from mild cognitive impairment (PD-MCI) to full-scale dementia (PDD). While it is well known that the risk of developing dementia increases as the disease progresses, the timing, profile, and rate of CI differs broadly within PD patients, which necessitates stratification and monitoring strategies. Autonomic dysfunction in PD involves thermoregulatory symptoms such as hyperhidrosis, hypohidrosis, and hypothermia (3). Thermoregulatory dysfunction in PD is known to involve the brainstem and hypothalamus with alpha-synuclein deposits (3). PD patients with symptoms of autonomic dysfunction display reduced functional connectivity in disrupted thalamo-striatal-hypothalamic circuits, which suggests a potential association with deficits in cognitive function (4). Moreover, autonomic dysfunction has been associated with functional brain connectivity as well as CI in *de novo* patients with PD (5). In addition, autonomic dysfunction was identified as a strong risk factor for declines in cognitive function or future development of dementia in patients with PD (6). Furthermore, a recent study reported a correlation between hyperhidrosis and CI (7). These accumulating findings suggest a close link between sweating disorders and CI. As a high-sweat excretion area where specific microbial taxa thrive due to changes in both humidity level and sweat composition, the underarm (axilla) is commonly affected by sweating disorders. Accordingly, we hypothesized that CI stages of PD patients may be tracked through alterations in the axillary microbiota composition.

In this study, we recruited 129 subjects (55 PD-MCI, 48 PDD, and 26 normal-cognition healthy controls [HC]) to characterize axillary microbiota profiles and assess whether there are microbiome signatures differentiating CI stages in PD.

## RESULTS

### Characteristics and statistics of study groups.

A total of 129 individuals, including 103 PD patients (55 PDD and 48 PD-MCI) and 26 HC, were included in the study. Demographics and clinical features of participants are summarized in Table 1. Hoehn and Yahr (HY) staging scale and Unified Parkinson’s Disease Rating Scale (UPDRS-II) scores of PD patients are shown in Table S1 in the supplemental material. The mean age differed significantly among three groups, while there was no significant difference in the proportion of females. We also found that education level differed significantly between PD patients and HC, while there was no significant difference between PDD and PD-MCI.

**TABLE 1:** Clinical and demographic features of the study cohort^*a*^

Characteristics	HC	PD-MCI	PDD
Number (*n*)	26	48	55
Age (yrs, mean ± SD)	59.9 ± 8.19	67.5 ± 9.3^*b*^	71.4 ± 7.8^*b*,^^*c*^
Sex (female, *n* [%])	14 (53.9)	21 (43.8)	25 (45.5)
Education (yrs, mean ± SD)	10.5 ± 4.9	6.3 ± 4.6^*b*^	4.7 ± 4.7^*b*^
MMSE	27.7 ± 1.8	23.9 ± 2.6^*b*^	18.3 ± 4.2^*b*,^^*c*^

a PD, Parkinson’s Disease; MMSE, Mini-Mental State Examination; HC, Healthy Control; PD-MCI, Parkinson’s Disease with Mild Cognitive Impairment; PDD, Parkinson’s Disease with Dementia.

b *P* < 0.05 for pairwise comparison with HC.

c *P* < 0.05 for pairwise comparison with PD-MCI.

**FIG 1:**
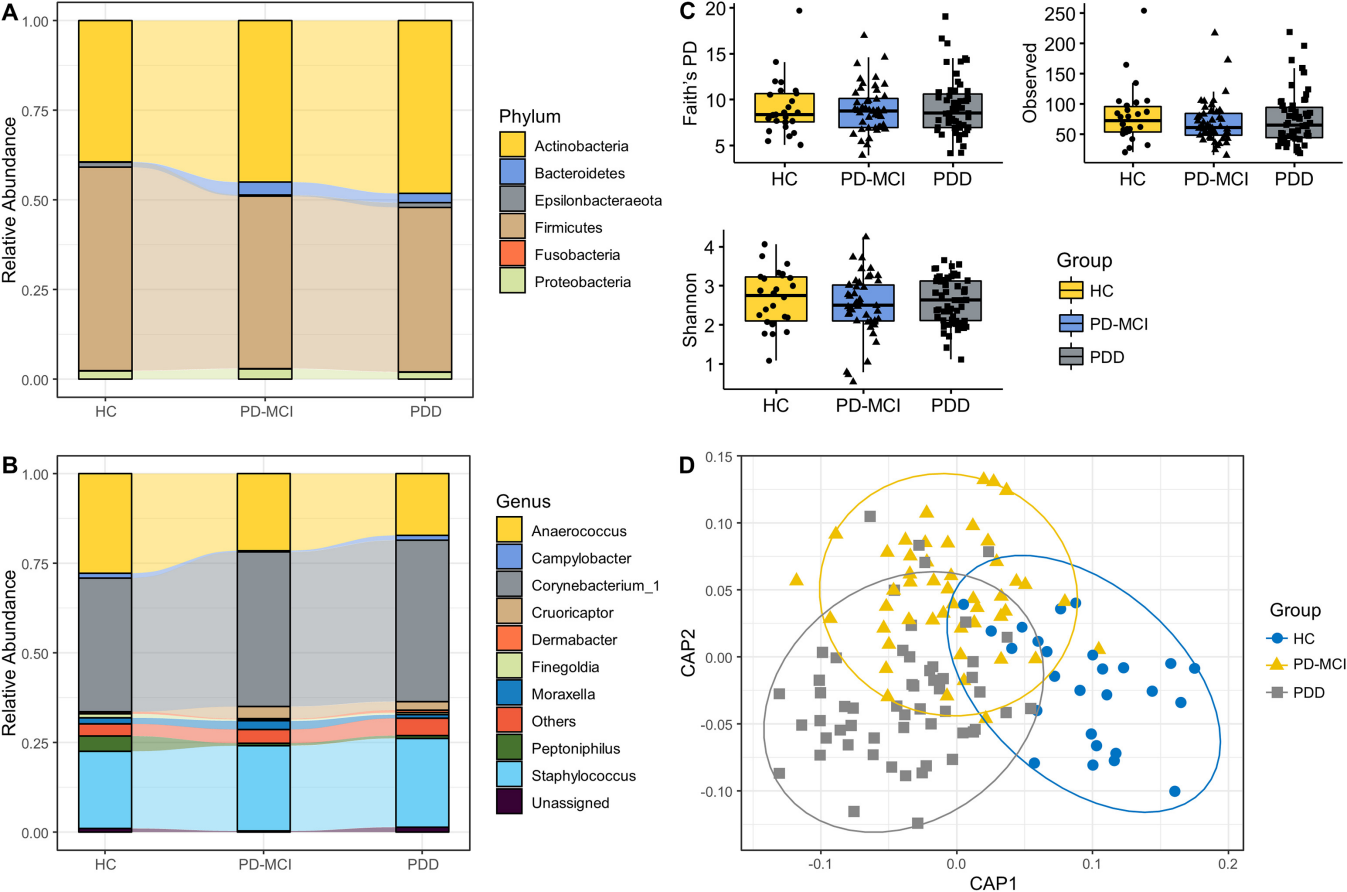
Overview of axillary microbiota composition and diversity across study groups. (A) The 10 most common phyla in axillary microbiota samples. (B) The 10 most common genera in axillary microbiota samples. Phyla and genera that were not among 10 most common taxa were grouped into “Others.” Each bar represents relative abundance distribution for a study group. (C) Alpha diversity comparisons of axillary microbiota samples between study groups. (D) CAP analysis of axillary microbiota samples. The coordinate table obtained from CAP analysis was imported into R environment and ggplot2 package was used to generate the CAP plot with ellipses.

The most abundant phyla in axillary microbiota across sample groups were *Actinobacteria*, *Bacteroidetes*, *Epsilonbacteraeota*, *Firmicutes*, *Fusobacteria*, *Patescibacteria*, *Proteobacteria*, *Spirochaetes*, *Synergistetes*, and *Tenericutes* (Fig. 1A). Among these, *Actinobacteria* and *Firmicutes* were dominant (>90% relative abundance) across all study groups. The three most abundant genera across study groups were *Corynebacterium*, Staphylococcus, and *Anaerococcus*, accounting for more than 80% of the bacterial community (Fig. 1B). The genera level abundances for each sample are shown in Fig. S1 in the supplemental material.

We want to note that the choice of variable regions and specific primers can miss some locally colonized important taxa (8). These biases have been discussed in several publications. For example, Meisel et al. (9) reported that 806R primer (10) used for amplification of V4 region is unable to detect *Cutibacterium* due to a “T” nucleotide at the 3′ end of primer. Conversely, our reverse primer includes additional “CC” nucleotides at the 3′ end after “T,” thus differing from the 806R reverse primer reported by Caporaso et al. (10). Clearly, the presence of “CC” at the 3′ end of 806R enables amplification of the marker gene from *Cutibacterium*, albeit the efficiency of these two primers may differ. Indeed, Zeeuwen et al. (11) compared the efficiency of these V4 reverse primers for amplification of *Cutibacterium* from skin samples and confirmed that 806R primer cannot amplify *Cutibacterium*, while 802R primer, which has additional “CC” at the 3′ end, can amplify *Cutibacterium*. In addition, Castelino et al. (12) showed that the V3-V4 region amplified with a reverse primer that ends identically to our reverse primer can accurately represent skin microbial communities. Therefore, we expect our choice of primers amplifying V3-V4 regions to have a limited impact, if any, on the results.

### Structural diversity measures.

There were no significant differences in alpha diversity indices between study groups (Fig. 1C). To examine the variation between samples in composition of axillary microbial communities, we generated a beta-diversity ordination using the Aitchison distance, which simply involves applying principal component analysis (PCA) to the centered log-ratio (CLR) transformed feature counts. We tested whether the samples cluster beyond what was expected by sampling variability while adjusting for the confounding effects of age, sex, and education. The results showed a significant difference between PD patients and HC (*P* = 0.042); however, when adjusted for potential confounders, this association was attenuated and not significant (*P* = 0.092). We have also used the Jaccard distance to test differences between study groups, which showed a significant separation between HC and PD patients (*P* = 0.033) but not between the three groups. We have also used canonical analysis of principal components (CAP) analysis to further examine the variation between samples (Fig. 1D). CAP analysis showed a significant separation between the three groups (*P* = 0.02, trace statistic = 0.969).

### Association of differentially abundant taxa with cognitive status.

**FIG 2:**
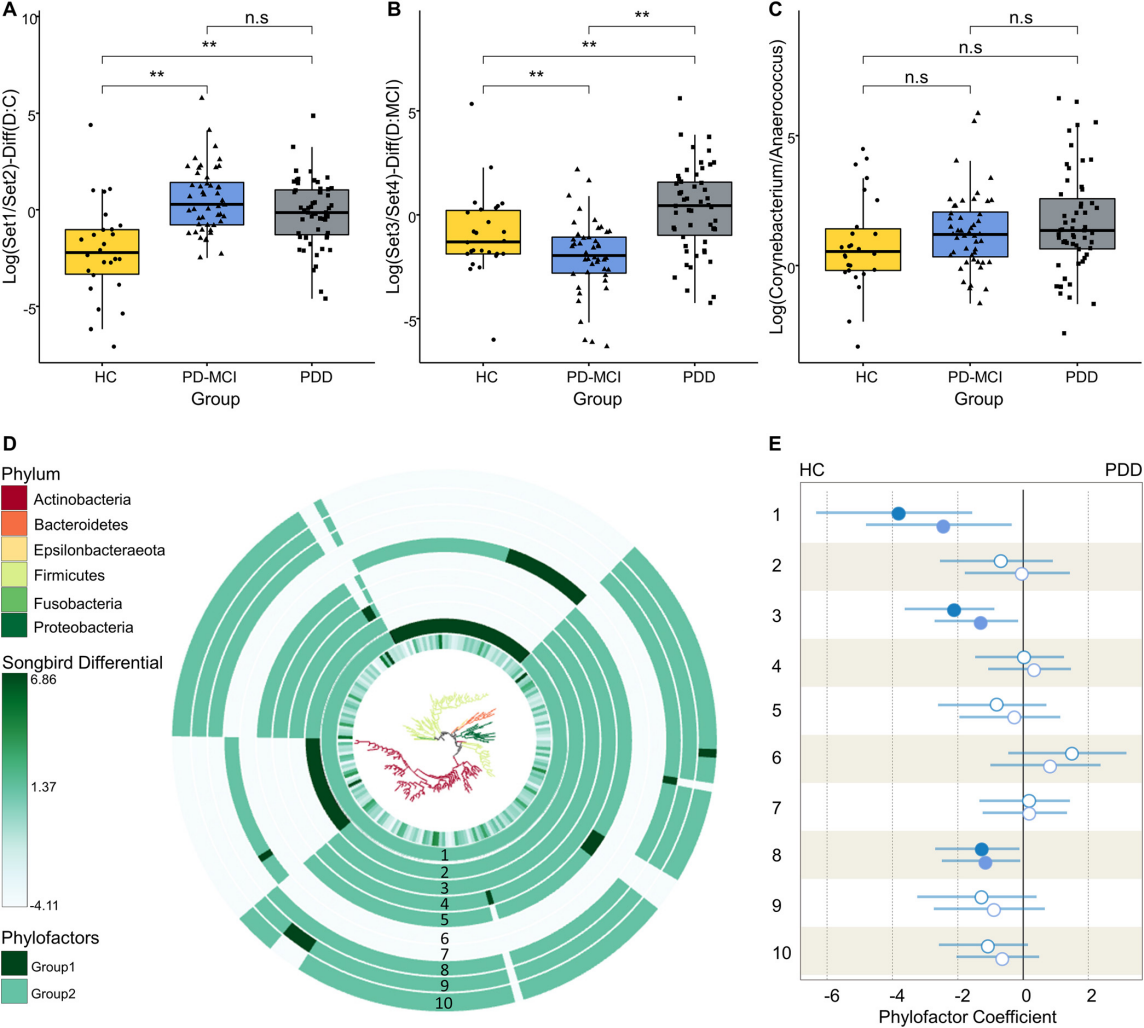
Differential ranking and phylogenetic factorization analysis of taxa associated with cognitive stages. (A) Boxplots of the log ratios of highest (Set1) and lowest (Set2) 25% ranked ASVs separating HC and PD-MCI groups. (B) Boxplots of the log ratios of highest (Set3) and lowest (Set4) 25% ranked ASVs separating PD-MCI and PDD groups. (C) Boxplots of the log ratios of *Corynebacterium* and *Anaerococcus* genera across study groups. Asterisk indicates statistical significance (*P* < 0.05). (D) EMPress plot showing phylogenetic tree with branches colored at phylum level. Tree was generated with only the ASVs used in differential ranking analysis. Innermost ring represents the estimated log-fold changes produced by Songbird. Outer bar plot rings indicate the first 10 Phylofactor-based phylogenetic partitions (phylofactors). Clades which are not included in each phylofactor appear light gray in the bar plot ring. (E) Regression coefficients predicted by the Phylofactor multivariate model for each phylofactor are shown in the forest plot. The forest plot to the right indicates the estimated increase in phylofactor associated with cognitive status, while the forest plot to the left shows estimated decrease in phylofactor associated with cognitive status. Error bars indicate 95% confidence intervals for the regression coefficients. Estimated coefficients for PD-MCI and PDD groups are shown in light and dark blue circles, respectively. Filled blue shapes indicate significance (*P* < 0.05) while empty circles indicate a nonsignificant association.

We have used Songbird to generate a multinomial regression model estimating differentially abundant taxa. To evaluate whether the model was overfit, we compared it against a baseline model and obtained a pseudo-Q2 value of 0.026, suggesting that the Songbird model was not overfit. Then, we generated log ratios and visualized the results using Qurro. Next, we computed the log ratio of the highest 25% and lowest 25% of the ranked amplicon sequence variant (ASVs) and found significant differences distinguishing HC from PD-MCI and PD-MCI from PDD groups (*t* test, *P* = 6.45e-05 for HC versus PD-MCI [Set1/Set2], *P* = 2.00e-03 for PD-MCI versus PDD [Set3/Set4]) (Fig. 2A and B). To determine which taxa are mainly responsible for these differences between groups, we examined the features enriched in the highest- and lowest-ranked ASV lists. We observed a trend of an increasing *Corynebacterium*/*Anaerococcus* ratio from HC to PDD; however, this trend did not reach statistical significance (Fig. 2C). On the other hand, we noticed a heterogenous distribution of *Corynebacterium* and *Anaerococcus* ASVs across the highest- and lowest-ranked taxa which differentiate PD-MCI and PDD from HC, suggesting a subgenus-level separation. We also used MaAsLin2 to explore associations of individual taxa with clinical and demographic variables while controlling for covariates and found that increased abundance of the Staphylococcus genus was associated with age while decreased abundance of the *W5053* genus was associated with PDD.

We have applied a random forest machine learning approach to predict CI stages of the study participants using microbiota profiles. The results were visualized using confusion matrix, per-class receiver operating characteristic (ROC) curves and a heatmap of the most important features which maximize model accuracy (Fig. S2). The confusion matrix revealed that the overall accuracy of the prediction was 54% compared to baseline accuracy (42%), indicating a low predictive performance (Fig. S2A). ROC analysis showed that the model had a better predictive performance for the HC (72%) and PDD (66%) groups, while predictive performance was lower for MCI group (45%), which may reflect MCI being a transitional stage from HC to PDD (Fig. S2B). The model revealed that the most important features maximizing the predictive accuracy belong to 14 different genera (Fig. S2C). Importantly, *Corynebacterium*, *Anaerococcus*, *Peptoniphilus*, and *W5053* were among these genera.

### Differential abundance of specific clades associated with CI.

We complemented our analysis by applying a phylogenetic partitioning of the taxa which differentiated the study groups. We generated 10 phylofactors using the default parameters optimized for explaining maximal variance while adjusting for potential confounders (Fig. 2D and E). Three of these factors were statistically significant separations between study groups. The first phylofactor suggested that a significant reduction of the *Anaerococcus* genus is associated with progression of CI (*P* = 0.025 for HC versus PD-MCI, *P* = 0.002 for HC versus PDD). The third phylofactor predicted that reduced abundance of the *Peptoniphilus* and *W5053* genera was associated with progression of CI (*P* = 0.029 for HC versus PD-MCI, *P* = 0.002 for HC versus PDD). The eighth phylofactor identified a single ASV belonging to the *Peptoniphilus* genus as being significantly less abundant in PD patients (*P* = 0.038 for HC versus PD-MCI, *P* = 0.035 for HC versus PDD).

### Functional profile and phenotype predictions.

**FIG 3:**
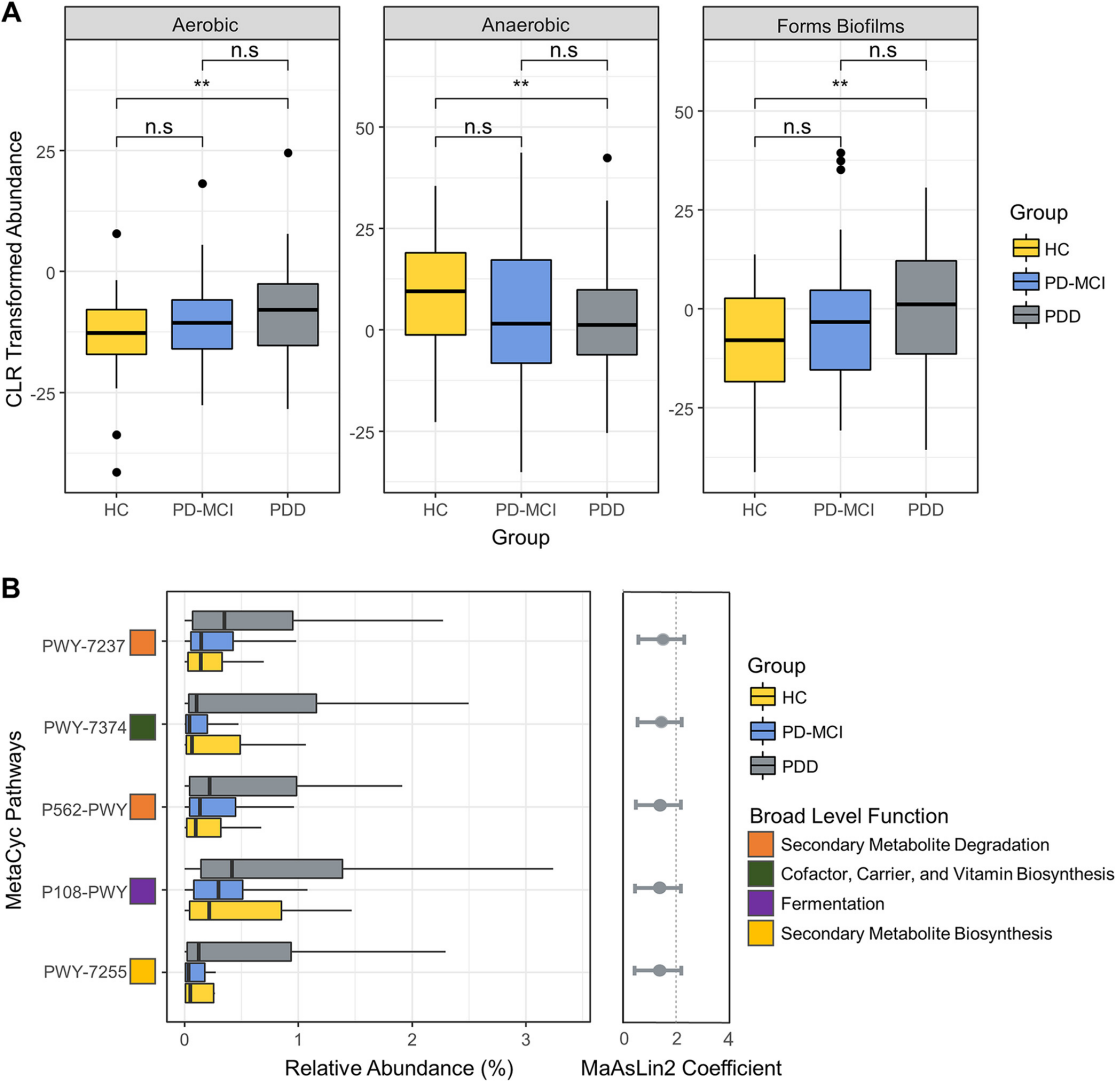
The phenotype and functional predictions profile predictions for axillary microbiota across study groups. (A) Bugbase-predicted phenotypes significantly associated with study groups. Asterisk indicates statistical significance (*P* < 0.05). (B) Relative abundance of the PICRUSt2-predicted MetaCyc pathways significantly associated with PDD group, and MaAsLin2-calculated coefficients for the associations of the predicted pathways with PDD.

BugBase predicted significant differences in richness of aerobic and anaerobic bacteria and biofilm formation potential between HC and PDD groups (Fig. 3A). There was a trend of an increased proportion of aerobic bacteria and a decreased proportion of anaerobic bacteria, which reached statistical significance between HC and PDD (*P* < 0.05). Biofilm formation potential was also found to increase with the progression of CI, reaching statistical significance (*P* < 0.05) between HC and PDD. Analyses of other bacterial phenotypes, namely, stress tolerance, the proportions of Gram-negative and Gram-positive bacteria, pathogenic potential, and contained mobile genetic elements, revealed no significant differences between groups.

Using PICRUSt2 and MaAsLin2, we found that 5 MetaCyc functional modules are significantly enriched with the progression of CI, including PWY-7237 (myo-, chiro-, and scillo-inositol degradation), PWY-7374 (1,4-dihydroxy-6-naphthoate biosynthesis I), P562-PWY (myo-inositol degradation I), P108-PWY (pyruvate fermentation to propionate I), and PWY-7255 (ergothioneine biosynthesis I). The results for these modules are shown at Fig. 3B. It should be noted that PICRUSt2 performs functional predictions using 16S rRNA gene amplicon sequences, thus, it may be biased toward the sequenced genomes and cannot reach enough resolution to differentiate strain-specific functionality (13). Thus, these functional prediction results should be interpreted with caution.

## DISCUSSION

In this study, we observed changes in the axillary microbiota in association with CI stages in PD, particularly an increasing trend in the log ratio of *Corynebacterium* to *Anaerococcus* with the progression of CI. Additionally, functional predictions showed elevated proportions of aerobic bacteria and biofilm formation capacity in the PD-MCI and PDD groups, and increases in myo-inositol degradation, ergothioneine biosynthesis, propionate biosynthesis, and menaquinone biosynthesis with the progression of CI.

We characterized the axillary microbiota of PD patients at different CI stages (PD-MCI and PDD) and identified *Corynebacterium*, Staphylococcus, and *Anaerococcus* as main components of the axillary microbiota across all study groups, which is consistent with previous studies on the axillary microbiota (14). There were no significant differences in alpha diversity between study groups. On the other hand, CAP analysis showed that groups were differentiated based on their axillary microbiota profiles. In addition, differential ranking analysis revealed that the differences in axillary microbiota profiles distinguish not only the PD-MCI group from HC, but also PDD from PD-MCI. Here, the ability of axillary microbiota to differentiate PD-MCI from HC is particularly important as it supports the potential of the axillary microbiota for early detection of PDD.

A variety of skin disorders have been associated with PD, particularly hyperhidrosis, with its characteristic odor. Armpits are known to be high sweat excretion areas where specific microbial taxa may thrive due to changes in both humidity level and sweat composition; for instance, *Corynebacterium* is known to favor moist areas and is associated with axillary malodor (15). Although we observed a trend of an increasing ratio of *Corynebacterium* to *Anaerococcus* with CI progression, these differences did not reach statistical significance; this is possibly due to further subgenus-level separations within these genera across study groups, which were observed in the differential ranking analysis. Thus, a more comprehensive strain-level profiling of *Corynebacterium* and *Anaerococcus* can provide better resolution and understanding of the changes in axillary microbiota with CI progression. Phylogenetic factorization also revealed significant decreases in the abundance of three genera belonging to *Clostridiales*, namely *Anaerococcus*, *Peptoniphilus*, and *W5053*, associated with PD-MCI and PDD. Decreased abundances of *Clostridiales* have been reported in the gut microbiota of Alzheimer’s disease and post-stroke cognitive impairment patients (16, 17). The depletion of *Clostridiales* in axillary microbiota with CI may reflect a result of the interactions in the gut-skin axis on microbiota members.

Phenotype predictions showed increases in the proportion of aerobic bacteria and in biofilm formation capacity. Total aerobic bacteria and increased biofilm formation capacity phenotypes have been associated with malodor intensity in axillary regions and skin disorders, respectively (18, 19). These predictions thus suggest potential associations of increased proportion of aerobic bacteria and biofilm formation capacity in axillary microbial communities with CI in PD.

Functional profile predictions also showed the enrichment of several pathways in association with worsening CI. Among these pathways, myo-inositol degradation and pyruvate fermentation to propionate I were particularly interesting. A previous study reported elevated levels of myo-inositol in the cerebrospinal fluid of patients with Alzheimer’s dementia (20). It is also well known that myo-inositol is present in human sweat (21); however, to the best of our knowledge, there have been no studies of skin myo-inositol levels associated with CI in PD patients. Our findings suggest a potential link between CI and accelerated myo-inositol degradation in axillary regions. Propionate is a short-chain fatty acid in humans produced by microbial fermentation from lactate and is one of the main components of human sweat (22). Skin microbiota members, specifically *Actinobacteria*, are known to be propionate producers. In addition, saliva, fecal, and hippocampus propionate levels have been found to increase significantly in Alzheimer’s disease patients (23). Our results also suggest a potential association between the increase in propionate biosynthesis by skin microbiota and CI stages in PD. Recently, one line of evidence (24–26) indicates that PD patients have intestinal inflammation and poor fecal short-chain fatty acid (SCFA) production, which correlates with onset of the disease. Therefore, modulating microbiota using diet and other means is likely to have a restorative effect on SCFA production. Interestingly, SCFAs produced by the skin microbiota and their role has attracted limited attention, whereas skin-generated SCFAs were also shown to downregulate inflammation in the skin as they do in the gut (27, 28). Considering that the gut and the skin are organs with crucial immune and neuroendocrine roles and bidirectional communication, future research directions should include integrated investigations of the effects of human gut and skin microbiota modulation, through diet and other means, on the management of non-motor symptoms in PD, including CI.

The main limitations of this study are as follows: (i) this was an exploratory study, so it was impossible to establish any causal relationships or reveal the mechanisms responsible for CI in PD; (ii) information regarding not using antiperspirants or deodorants and excessive sweating was self-reported, thus potentially unreliable for some subjects, (iii) a lack of control for other confounding factors such as BMI, diet, and medicine; (iv) there may be potential biases arising from primer selection in microbial community profiles, just as in any other amplicon-based microbiota study; and (v) functional predictions based on 16S rRNA sequencing data have inherent limitations.

## MATERIALS AND METHODS

The study was approved by the ethics committee of the Istanbul Medipol University with authorization number 10840098-604.01.01-E.3958, and informed consent was obtained from all participants.

### Study subjects and clinical characteristics.

A total of 129 subjects (HC, *n* = 26; PD-MCI, *n* = 48; PDD, *n* = 55) were recruited at two health centers, the Medipol University Training Hospital and the Bakirkoy Research and Training Hospital for Psychiatric and Neurological Diseases. Clinical and demographic information, including age, sex, and years of education, were collected during clinic visits. The patients were examined by experienced neurologists and PD diagnoses were made within the framework of the “United Kingdom Parkinson’s Disease Society Brain Bank” criteria. Subjects with previous head trauma, stroke, or exposure to toxic substances, and those with symptoms suggestive of Parkinson’s plus syndromes, were excluded from the study. The Hoehn-Yahr scale of Parkinson’s stages was used to determine the stage of the disease, and The Movement Disorder Society’s diagnostic criteria for Parkinson’s Disease Dementia were used for dementia evaluation. The diagnosis of MCI was made within the framework of the previously defined criteria (29). HC were collected considering the demographic characteristics of the patient groups.

### Sampling and DNA extraction.

Axillary microbiota was sampled using sterile hydraflock swabs (Puritan Medical Products, Guilford, ME, USA) in the clinic. Participants were asked not to shower and to stop using any antiperspirant or deodorant products, if any, for a minimum of 24 h before their clinic visit. In brief, sterile swabs were first placed in a storage buffer containing 1 mL Tris-ethylenediaminetetraacetic acid (TE) buffer with 10% glycerol and then rubbed on the axillae area for 10 s. After sample collection, the swab tips were cut off with sterilized scissors and placed back into the storage buffer in the Eppendorf tubes. The samples were immediately transferred to −80°C freezer for storage until further processing.

Microbial DNA extraction from axillary samples was performed using a DNeasy PowerSoil (Qiagen, Hilden, Germany) with modifications to the manufacturer's protocol. In brief, swabs were transferred from storage buffer to the PowerBead tube. Storage buffer was centrifuged at 10,000 × *g* for 5 min, supernatant was discarded, and the pellet was resuspended with 400 μL bead-beating buffer and transferred to the PowerBead tube. Samples were homogenized by bead-beating using a Next Advance Bullet Blender (30 s at level 4, 30 s incubation on ice, and 30 s at level 4). After the bead-beating step, the manufacturer's protocol was followed without any modification.

### Library preparation and sequencing.

The V3-V4 regions of 16S rRNA gene were amplified with F-5′-CCTACGGGNGGCWGCAG-3′ and R-5′-GACTACHVGGGTATCTAATCC-3′ universal bacterial primers. Amplicon libraries were prepared following Illumina’s 16S Metagenomic Sequencing library preparation protocol and sequenced using a MiSeq platform and 2 × 250 paired-end kit. A total of 129 samples were sequenced, along with an extraction negative control and a no-template PCR control, per run.

### Analysis of axillary microbiota.

Raw sequencing data were analyzed using the Nephele platform (v.1.6, http://nephele.niaid.nih.gov) (30) using the SILVA v.132 database (31). The contaminant sequences were identified and removed using the *decontam* package (32) based on negative-control samples. Only ASVs present in at least 10 samples were included in the downstream analyses. Samples were rarefied to minimum sampling depth (10,342 reads) before alpha and beta diversity analyses. However, no rarefaction was performed before using other tools, such as Songbird, MaAsLin2, and Phylofactor, as these tools include internal normalization steps and do not require prior rarefaction. Diversity analyses were performed using QIIME2 (33) and phyloseq (34), while differential abundance analysis of taxa associated with CI stage was conducted using Songbird (35) with the following parameters: formula: “Age+Batch+MMSE+Sex+CDR+Education+C(Group, Diff, levels=['HC', 'MCI', 'PDD']),” –p-epochs 10000 –p-differential-prior 0.5 –p-summary-interval 7. The results were visualized using Qurro (36). In addition, a random forest machine learning model was generated to predict CI stages. A total of 80% of samples were used to train the model and 20% were used for testing. The estimator parameter was set to 100. Furthermore, MaAsLin2 (37) was used to examine potential associations between the genera detected in axillary samples and metadata. Phylofactor (38) was used to identify abundance-based phylogenetic partitioning between clades through their associations with study groups. PICRUSt2 (13) was employed to predict the functional potential of the axillary microbial communities, and differentially abundant functional modules associated with CI stages were determined using MaAsLin2. Moreover, the potential microbiota phenotypes were predicted by BugBase pipeline (39). EMPress (40), *forestplot*, and *ggplot2* (41) R packages were used for visualizations.

### Statistical analysis.

Statistical analyses were conducted in R 3.6.1. A Kruskal-Wallis test was used for alpha diversity comparisons. Adonis, an implementation of permutational multivariate analysis of variance (PERMANOVA) from the vegan package, was used for beta diversity comparisons, with adjustment for potential confounding factors. A paired *t* test was used for continuous variables, namely age and education, while Fisher’s exact test was used for categorical variables. Differential ranking analysis was performed using Songbird, employing Welch's *t* test to determine statistical significance. Pairwise Mann-Whitney-Wilcoxon tests were performed for the comparison of BugBase predictions.

### Data availability.

The raw sequence data produced in this study have been deposited in the NCBI Sequence Read Archive database under accession no. PRJNA761243.
