# Supplementary material for: Axillary Microbiota Is Associated with Cognitive Impairment in Parkinson’s Disease Patients
Source: Microbiol Spectr. 2022 Feb 9;10(1):e02358-21. doi: 10.1128/spectrum.02358-21 (PMC8826741; doi:10.1128/spectrum.02358-21)
Supplement: SUPPLEMENTAL FILE 1 — Supplemental material. Download SPECTRUM02358-21_Supp_1_seq6.pdf, PDF file, 0.5 MB [file spectrum02358-21_supp_1_seq6.pdf]

Table S1. Hoehn and Yahr (HY) staging scale and Unified Parkinson's Disease Rating Scale (UPDRS-II) scores of PD patients

Patient ID	HY	UPDRS-II
701	1.5	19
703	1	18
704	-	-
708	1.5	26
709	2	45
710	2	33
711	1.5	27
712	2	30
713	1.5	45
714	-	29
715	4	58
716	2	21
717	1.5	28
718	3	79
719	2.5	57
720	2.5	37
721	3	28
722	2	15
724	3	64
726	2	38
727	2	52
730	4	20
734	4	97
735	1.5	19
737	1	14
740	1	37
743	2.5	41
744	3	64
746	-	-
747	3	28
748	1.5	23
757	2	43
758	3	75
761	2	61
766	1.5	33
767	3	46
768	2	53

770	1	33
772	3	25
773	4	49
774	4	79
777	2.5	48
778	3	62
779	1.5	26
782	1	39
783	2	45
784	1	10
786	3	47
787	4	63
789	2.5	20
790	1	15
793	-	-
796	-	-
797	3	54
802	4	43
803	3	42
804	4	51
805	3	58
807	1.5	45
808	1.5	41
809	1	46
810	1.5	32
827	2	57
831	1	18
832	4	76
833	2.5	58
834	3	39
836	1.5	29
839	3	48
841	1	18
842	2	73
843	2	64
846	1.5	28
847	2	29
848	4	65
851	3	49
852	2.5	80
853	1.5	25

858	2	32
859	2.5	52
860	1	19
861	1	20
872	3	47
873	3	59
876	2	47
877	1.5	34
883	1.5	27
885	4	58
886	2	21
888	4	78
893	1.5	42
902	2	49
903	2	45
906	3	43
907	1	23
908	3	42
909	2	31
911	2.5	55
912	1.5	30
913	2.5	70
914	1	21
915	4	58
916	1.5	35

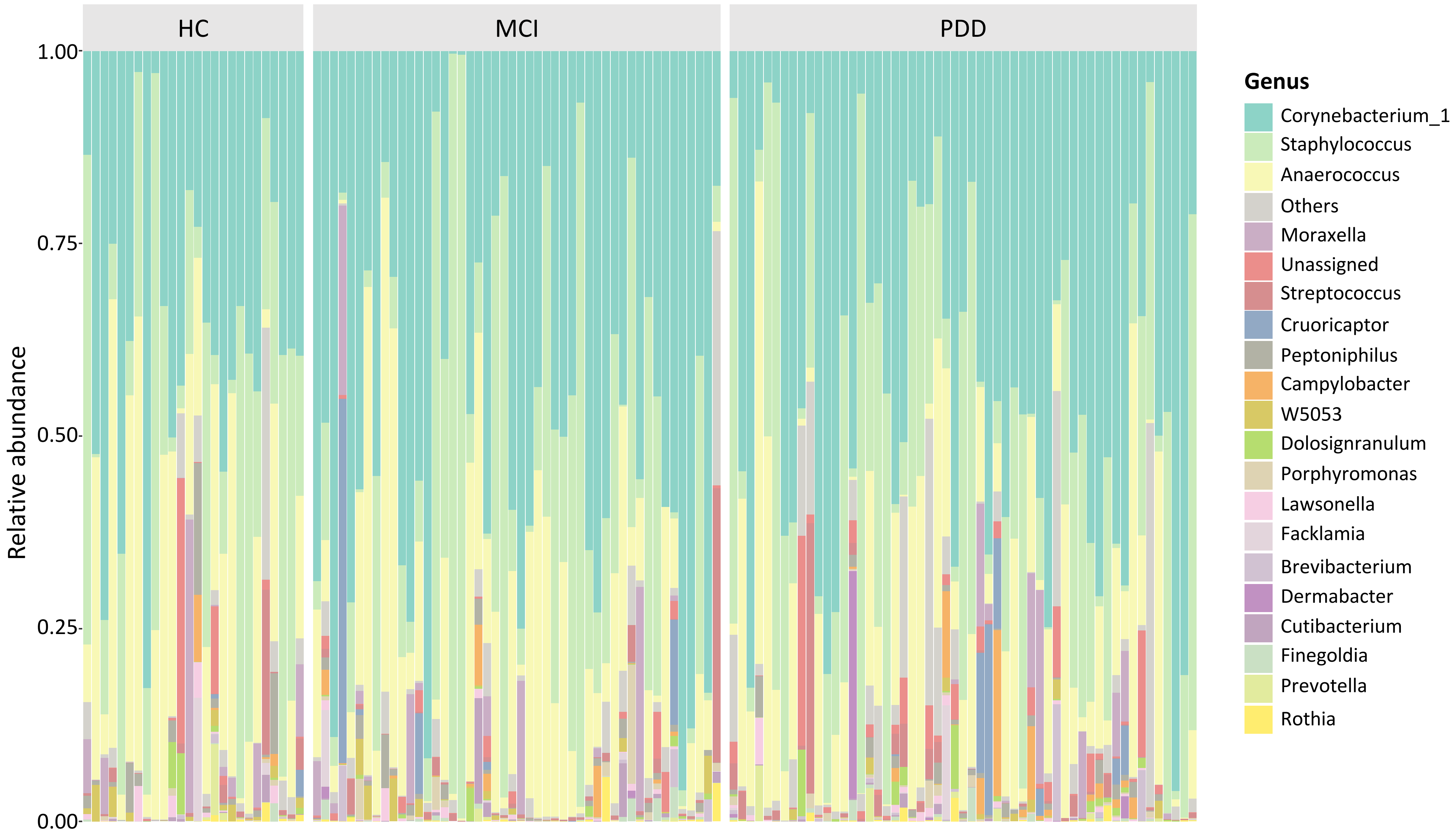

Figure S1. The genus level abundances in axillary microbiota samples. 20 most common genera in axillary microbiota samples. The genera that were not among 20 most common taxa were grouped into "Other." Each bar represents relative abundance distribution for a study subject.

A

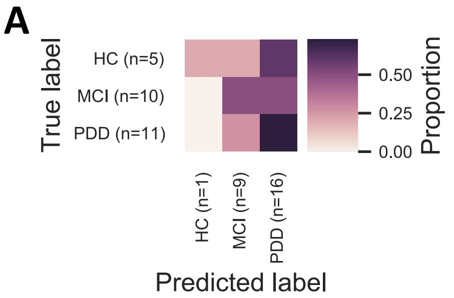

	HC	MCI	PDD	Overall Accuracy
**HC**	0.2	0.2	0.6	
**MCI**	0	0.5	0.5	
**PDD**	0	0.272727273	0.72727273	
**Overall Accuracy**				0.538461538
**Baseline Accuracy**				0.423076923
**Accuracy Ratio**				1.272727273

B

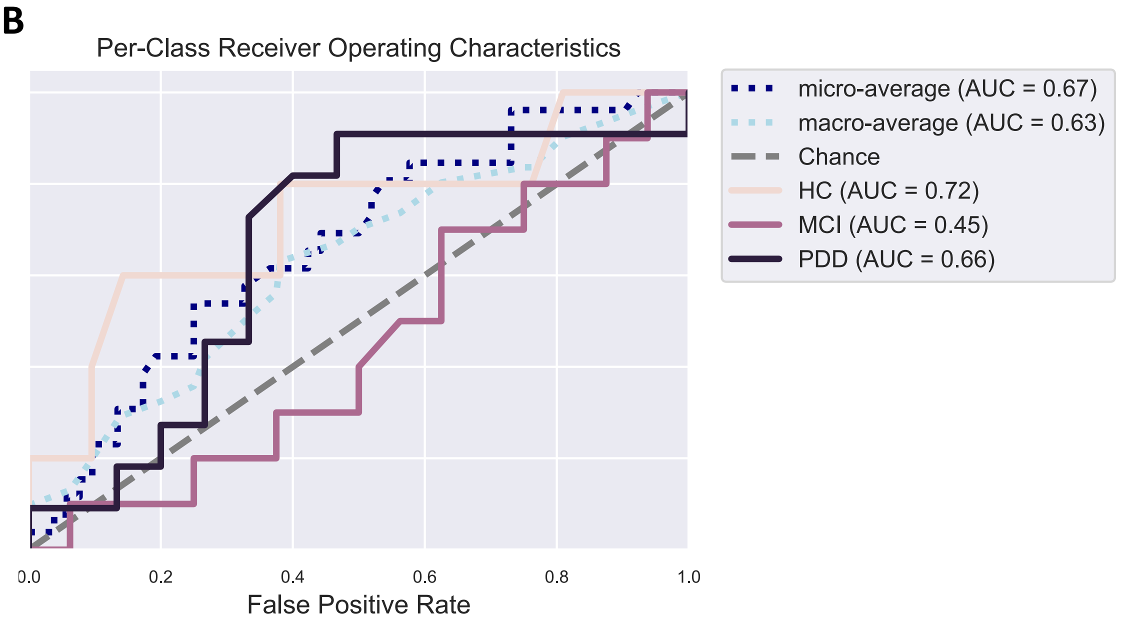

C

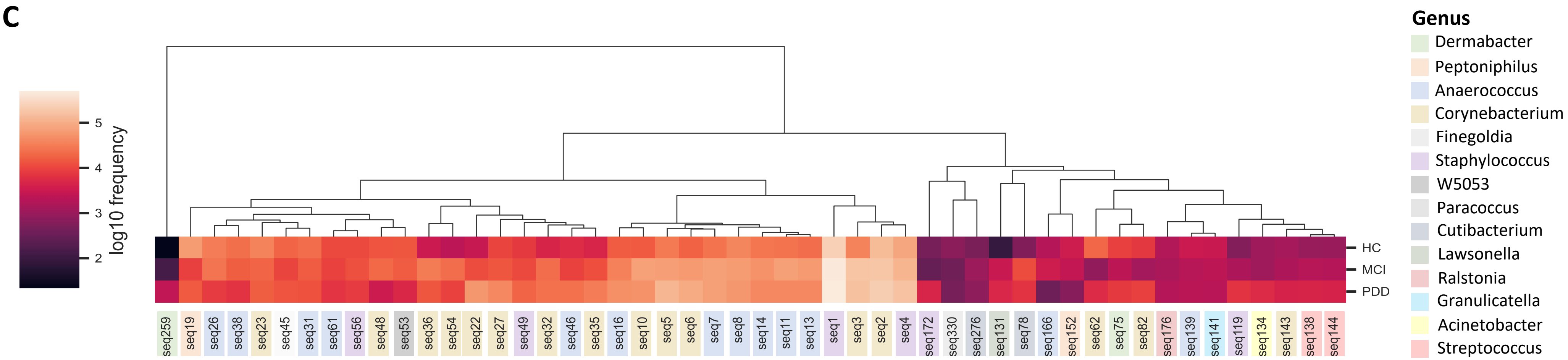

Figure S2. Prediction of CI stages using a random forest machine learning model. A. Confusion matrix and accuracy results, B. Per-class receiver operating characteristic (ROC) curves, C. The heatmap of the features that maximize the model accuracy.
